# ﻿Two new pestalotioid fungi from tropical fruits in Iran

**DOI:** 10.3897/mycokeys.115.136469

**Published:** 2025-03-20

**Authors:** Amirreza Amirmijani, Adel Pordel, Kowsar Dehghani, Mohammad Javad Pourmoghaddam, Hossein Masigol, Hans-Peter Grossart

**Affiliations:** 1 Department of Plant Protection, Faculty of Agriculture, University of Jiroft, Jiroft, Iran University of Jiroft Jiroft Iran; 2 Plant Protection Research Department, Baluchestan Agricultural and Natural Resources Research and Education Center, AREEO, Iranshahr, Iran Plant Protection Research Department, Baluchestan Agricultural and Natural Resources Research and Education Center Iranshahr Iran; 3 Department of Plant Protection, Faculty of Agricultural Sciences, University of Guilan, Rasht, Iran University of Guilan Rasht Iran; 4 Department of Plankton and Microbial Ecology, Leibniz Institute of Freshwater Ecology and Inland Fisheries (IGB), Alte Fischerhuette 2, 16775 Stechlin, Germany Department of Plankton and Microbial Ecology, Leibniz Institute of Freshwater Ecology and Inland Fisheries (IGB) Stechlin Germany; 5 Institute of Biochemistry and Biology, Potsdam University, Maulbeerallee 2, 14469 Potsdam, Germany Potsdam University Potsdam Germany

**Keywords:** Fungal diversity, *Mangiferaindica*, Pestalotioid fungi, *Psidiumguava*, Tropical fruits, two new species

## Abstract

In a survey of tropical plant diseases in southern and southeastern Iran, samples of diseased *Mangiferaindica* and *Psidiumguava* leaves with necrotic symptoms were collected between 2021 and 2022. Six representative isolates of *Neopestalotiopsis* and *Robillarda* (three isolates for each) were studied using morphological characteristics as well as multi-locus phylogenetic analysis based on (i) the internal transcribed spacer (ITS) region of the nuclear rDNA, (ii) part of the translation elongation factor 1-alpha (*tef1*), and (iii) the β-tubulin (*tub2*). After morphological investigation, our phylogenetic analysis revealed that the *Neopestalotiopsis* and *Robillarda* isolates under study differed from all previously described species within these genera. Based on our polyphasic approach, two new species, including *Neopestalotiopsisguava***sp. nov.** from necrotic *Mangiferaindica* and *Robillardakhodaparastii***sp. nov.** from *Psidiumguava* are described and illustrated from Iran.

## ﻿Introduction

Tropical fruits provide essential nutrition and serve as a source of income for farmers engaged in export production. The major cultivation areas for tropical fruits are predominantly in developing countries, particularly in Asia and Latin America (FAO 2020). Global trade in tropical fruits has expanded to unprecedented levels in recent years, reaching an aggregate export volume of nearly 9 million tons in 2023. Strong demand from key importing countries has driven significant investments in productivity improvements and expansion of cultivation areas in supplying countries, notably for avocados (FAO 2024). Iran is a major producer of fruits, including tropical fruits, due to favorable meteorological conditions, diverse climates, and significant temperature differences between the northern and southern regions (Saboki et al. 2012). Regarding tropical fruit production, the Provinces of Hormozgan and Sistan and Baluchistan, located in the south and southeast of Iran, are considered the most suitable regions due to their proximity to the equator, the Oman Sea, and the Indian Ocean (Saboki et al. 2012, 2014).

Pestalotioid fungi are diverse and exhibit various lifestyles, including plant pathogens, endophytes, and saprophytes (Crous et al. 2015a; Sun et al. 2023). Despite uncertainties regarding the generic divisions among pestalotioid fungi, the classification system based on the number of conidia cells in different genera is still in use (Shu et al. 2020). The genus *Pestalotia* was initially described by De Notaris in 1841 (Jiang et al. 2022). Subsequently, based on the number of conidia cells, *Pestalotia* was divided into four distinct genera: *Truncatella* (4 cells), *Pestalotiopsis* (5 cells), *Pestalotia* (6 cells), and *Monochaetia* (5 cells) (Sutton 1980). Phylogenetic analyses have validated Sutton’s classification based on morphological characteristics; however, species previously classified under *Pestalotia* have been reassigned to the genus *Seiridium* (Marin-Felix et al. 2019). Additionally, the genus *Pestalotiopsis* has been further categorized into *Pestalotiopsis* sensu stricto, *Neopestalotiopsis*, and *Pseudopestalotiopsis* (Maharachchikumbura et al. 2016). *Neopestalotiopsis* is distinct from *Pestalotiopsis*, characterized by the presence of versicolourous median cells (Jiang et al. 2022).

The genus *Robillarda* was introduced by Saccardo in 1880 to accommodate the type species *R.sessile* (Saccardo 1880). *Robillarda* is a pestalotioid fungus within the family Sporocadaceae, and characterized by conidia with appendage-bearing (Liu et al. 2019). Although the genus includes 41 morphological species, sequence data are available for only some of them (Liu et al. 2019; https://www.indexfungorum.org/names/Names.asp/, accessed 31 Aug 2024).

In the present study, leaf spots on *Mangifera* and *Psidium* were observed in Sistan and Baluchestan Province, Iran. Based on modern taxonomic approaches, we identified two new species of *Neopestalotiopsis* and *Robillarda* from Iran. Detailed morphological descriptions, illustrations, and phylogenetic information are provided here.

## ﻿Materials and methods

### ﻿Sampling and fungal isolation

During a survey conducted on tropical and subtropical fruit trees in the summer of 2021, a total of seventy-five samples were collected from mango (*M.indica*) plants displaying symptoms of leaf spot disease. The leaf samples were specifically gathered from various districts in the Provinces of Hormozgan (Siaho district) and Sistan and Baluchestan (Nikshahr, Ghasreghand, Rask, and Konarak districts), which are located in the southern and southeast regions of Iran, respectively. The infected samples were transported to the laboratory and stored in a refrigerator under dry conditions at a temperature of 4 °C. To begin the isolation process, the infected tissues were cut into 7–8 mm pieces, surface–disinfected with a 2% sodium hypochlorite solution for 3 min, rinsed twice with sterile distilled water, dehydrated, and subsequently placed on 2% water-agar (2% WA) in Petri dishes. These Petri dishes were maintained at 25 °C under alternating near-UV light and dark conditions (12 h light/12 h dark) for 7 days. After 48 h, conidia were observed growing on the leaf pieces and transferred to 2% WA using the single-spore method. Hyphal tips emerging from individual conidia were further transferred to a potato dextrose agar (PDA) medium to establish pure cultures (Refaei et al. 2011).

### ﻿Morphological characterization

Mycelia plugs were extracted from the purified colony and placed on PDA to assess the colony’s overall characteristics. Subsequently, the plugs were incubated at 25 °C under alternating near-UV light and dark conditions. After 7–10 d, the color of the colony and the conidial mass were documented. To further analyze the morphological features of the conidiomata and conidia, more than 200 conidiophores and conidia were examined using slide mounts prepared with lactophenol and lactophenol cotton blue. Morphometrical analyses were also conducted on 200 conidiophores and conidia. For this purpose, a BH2 Olympus light microscope (Japan) equipped with a Microbin 12MP USB2.0 camera was utilized. The holotype and ex-type specimens have been deposited in the Herbarium of the Mycology Laboratory at the University of Jiroft, Jiroft, Iran (UJFCC).

### ﻿DNA extraction, PCR, and sequencing

DNA was extracted from seven-day-old fungal mycelium using the protocol described by Zhong and Steffenson (2001). The entire internal transcribed spacer (ITS1-5.8S-ITS2) regions of the rDNA, the partial translation elongation factor 1- alpha (*tef1*) gene and b-tubulin (*tub2*) gene were amplified using the primer pairs ITS1 (5”-TCCGTAGGTGAACCTGCGG-3”) and ITS4 (5”-TCCTCCGCTTATTGATATGC-3”) (White et al. 1990), EF1-728F (5”-CATCGAGAAGTTCGAGAAGG-3”) and EF2 (5”-GGARGTACCAGTSATCATGTT-3”) (Carbone and Kohn 1999; O’Donnell et al. 1998), as well as Bt2a (5”-GGTAACCAAATCGGTGCTGCTTTC-3”) and Bt2b (5”-ACCCTCAGTGTAGTGACCCTTGGC-3”) (Glass and Donaldson 1995). PCR amplifications were carried out in a final volume of 25 μL. The PCR mixtures contained 10 μL of master mix (CinnaGen, Iran), which included 10 × PCR buffer, MgCl_2_, dNTPs, Taq DNA Polymerase, 11 μL of double-distilled water, 1 μL of each forward and reverse primers (10 pmol), and 2 μL of template DNA. The PCR amplifications were done using a thermocycler with the following thermal conditions for ITS: initial denaturation at 94 °C for 3 min, followed by 35 cycles of denaturation step at 94 °C for 30 s, annealing at 55 °C for 30 s, and extension at 72 °C for 30 s, and terminated with a final extension step at 72 °C for 10 min; for *tef1*: initial denaturation at 94 °C for 8 min, and then followed by 35 cycles each with denaturation at 94 °C for 15 sec, annealing at 55 °C for 20 sec and the extension at 72 °C for 1 min, and a final extension at 72 °C for 5 min; for *tub2*: initial denaturation at 94 °C for 3 min, and then followed by 35 cycles each with denaturation at 95 °C for 30 s, annealing at 53 °C for 30 s and the extension at 72 °C for 45 s, and a final extension at 72 °C for 90 s. All amplicons were sent to the Codon Genetic Group (Tehran, Iran) for sequencing.

### ﻿Phylogenetic analyses

To identify closely related taxa, BLASTn searches were done separately for the three loci. Type and reference sequences of related taxa were retrieved from the National Center for Biotechnology Information (**NCBI**), if available, based on recent publications on the genera *Neopestalotiopsis* (Maharachchikumbura et al. 2012, 2014a, 2014b; Fiorenza et al. 2022; Razaghi et al. 2024) and *Robillarda* (Crous et al. 2015a; Liu et al. 2019). All alignments were produced using the server versions of MAFFT v. 7.490 (http://mafft.cbrc.jp/alignment/server/; Katoh et al. 2019) and were manually checked and refined with MEGA Ver. 7 (Kumar et al. 2016). Following the results of BLASTn searches for generated sequences of the three loci (ITS, *tef1*, *tub2*), a phylogenetic analysis was performed for *Neopestalotiopsis* species including 91 isolates. Similarly, a phylogenetic placement was conducted for *Robillarda*, including 11 isolates. *Pestalotiopsiscolombiensis* and *P.diversiseta* were selected as the outgroup taxa for both trees (Table 1). After excluding ambiguously aligned and gappy regions, the resulting combined data matrix contained 1363 alignment positions across all three loci (494 from ITS, 471 from *tef1*, and 398 from *tub2*) for *Neopestalotiopsis* and 1306 alignment positions (518 from ITS, 443 from *tef1*, and 345 from *tub2*) for *Robillarda*.

**Table 1.** Isolation and accession numbers of sequences used in the phylogenetic analyses. Isolates/sequences in bold were isolated/sequenced in present study. N/A: not available. ^1^ T indicates ex-type material.

Species	Strain^1^	Origin	GenBank accession numbers			References
			ITS	*tef1*	*tub2*	
*Neopestalotiopsisacrostichi*	MFLUCC 17-1754^T^	Thailand	MK764272	MK764316	MK764338	Norphanphoun et al. (2019)
*Neopestalotiopsisalpapicalis*	MFLUCC 17-2544^T^	Thailand	MK357772	MK463547	MK463545	Kumar et al. (2019)
*Neopestalotiopsisaotearoa*	CBS 367.54^T^	New Zealand	KM199369	KM199526	KM199454	Maharachchikumbura et al. (2012)
*Neopestalotiopsisasiatica*	MFLUCC 12-0286^T^	China	JX398983	JX399049	JX399018	Maharachchikumbura et al. (2012)
*Neopestalotiopsisaustralis*	CBS 114159^T^	Australia	KM199348	KM199537	KM199432	Maharachchikumbura et al.(2014b)
*Neopestalotiopsisbrachiate*	MFLUCC 17-1555^T^	Thailand	MK764274	MK764318	MK764340	Norphanphoun et al. (2019)
*Neopestalotiopsisbrasiliensis*	COAD 2166^T^	Brazil	MG686469	MG692402	MG692400	Bezerra et al. (2018)
*Neopestalotiopsiscavernicola*	KUMCC 20-0269^T^	China	MW545802	MW550735	MW557596	Liu et al. (2021)
*Neopestalotiopsisceltidis*	CGMCC 3.23513^T^	China	OR247900	OR361449	OR381049	Razaghi et al. (2024)
*Neopestalotiopsischrysea*	MFLUCC 12-0261^T^	China	JX398985	JX399051	JX399020	Maharachchikumbura et al. (2012)
*Neopestalotiopsisclavispora*	MFLUCC 12-0281^T^	China	JX398979	JX399045	JX399014	Maharachchikumbura et al. (2012)
*Neopestalotiopsiscoffeae-arabicae*	HGUP4019^T^	China	KF412649	KF412646	KF412643	Song et al. (2013)
*Neopestalotiopsisconcentrica*	CFCC 55162^T^	China	OK560707	OM622433	OM117698	Peng et al. (2022)
*Neopestalotiopsiscubana*	CBS 600.96^T^	Cuba	KM199347	KM199521	KM199438	Maharachchikumbura et al.(2014b)
*Neopestalotiopsisdendrobii*	MFLUCC 14-0106^T^	Thailand	MK993571	MK975829	MK975835	Ma et al. (2019)
*Neopestalotiopsisdolichoconidiophora*	CGMCC 3.23490^T^	China	OR247911	OR361421	OR381021	Razaghi et al. (2024)
*Neopestalotiopsisegyptiaca*	CBS 140162^T^	Egypt	KP943747	KP943748	KP943746	Crous et al. (2015b)
*Neopestalotiopsiselaeagni*	HGUP10002 ^T^	China	MW930716	MZ203452	MZ683391	He et al. (2022)
*Neopestalotiopsiselaeidis*	MFLUCC 15-0735^T^	Thailand	ON650690	ON734012	N/A	Konta et al. (2023)
*Neopestalotiopsisellipsospora*	MFLUCC 12-0283^T^	China	JX398980	JX399047	JX399016	Maharachchikumbura et al. (2012)
*Neopestalotiopsiseucalypticola*	CBS 264.37^T^	N/A	KM199376	KM199551	KM199431	Maharachchikumbura et al.(2014b)
*Neopestalotiopsiseucalyptorum*	CBS 147684^T^	Portugal	MW794108	MW805397	MW802841	Diogo et al. (2021)
*Neopestalotiopsisfoedans*	CGMCC 3.9123^T^	China	JX398987	JX399053	JX399022	Maharachchikumbura et al. (2012)
*Neopestalotiopsisformicarum*	CBS 362.72^T^	Ghana	KM199358	KM199517	KM199455	Maharachchikumbura et al.(2014b)
*Neopestalotiopsisfragariae*	ZHKUCC 22- 0113^T^	China	ON553410	ON569076	ON569075	Prematunga et al. (2022)
*Neopestalotiopsisguajavae*	FMBCC 11.1	Pakistan	MF783085	MH460868	MH460871	Ul Haq et al. (2021)
*Neopestalotiopsisguangxiensis*	CGMCC 3.23505^T^	China	OR247881	OR361440	OR381040	Razaghi et al. (2024)
***Neopestalotiopsisguava***	**UJFCC2084^T^**	**Iran**	**PP038121**	**PP053741**	**PP053735**	**This study**
***Neopestalotiopsisguava***	**UJFCC2085**	**Iran**	**PP038120**	**PP053740**	**PP053734**	**This study**
***Neopestalotiopsisguava***	**UJFCC2086**	**Iran**	**PP038122**	**PP053742**	**PP053736**	**This study**
*Neopestalotiopsishaikouensis*	SAUCC212271^T^	China	OK087294	OK104877	OK104870	Hsu et al. (2024)
*Neopestalotiopsishispanica*	CBS 147686^T^	Portugal	MW794107	MW805399	MW802840	Diogo et al. (2021)
*Neopestalotiopsishonoluluana*	CBS 114495^T^	USA	KM199364	KM199548	KM199457	Maharachchikumbura et al.(2014b)
*Neopestalotiopsishydeana*	MFLUCC 20-0132^T^	Thailand	MW266069	MW251129	MW251119	Huanlauek et al. (2021)
*Neopestalotiopsisiberica*	CBS 147688^T^	Portugal	MW794111	MW805402	MW802844	Diogo et al. (2021)
*Neopestalotiopsisiranensis*	CBS 137768^T^	Iran	KM074048	KM074051	KM074057	Ayoubi and Soleimani (2016a)
*Neopestalotiopsisjavaensis*	CBS 257.31^T^	Indonesia	KM199357	KM199543	KM199437	Maharachchikumbura et al.(2014b)
*Neopestalotiopsisketeleeriae*	MFLUCC 13-0915^T^	China	KJ023087	KJ023089	KJ023088	Song et al. (2014)
*Neopestalotiopsislongiappendiculata*	CBS 147690^T^	Portugal	MW794112	MW805404	MW802845	Diogo et al. (2021)
*Neopestalotiopsismacadamiae*	BRIP 63737c^T^	Australia	KX186604	KX186627	KX186654	Akinsanmi et al. (2017)
*Neopestalotiopsismaddoxii*	BRIP 72266a^T^	Australia	MZ303782	MZ344167	MZ312675	Prasannath et al. (2021)
*Neopestalotiopsismegabetaspora*	CGMCC 3.23474^T^	China	OR247875	OR361410	OR381010	Razaghi et al. (2024)
*Neopestalotiopsismesopotamica*	CBS 336.86^T^	Turkey	KM199362	KM199555	KM199441	Maharachchikumbura et al.(2014b)
*Neopestalotiopsismianyangensis*	CGMCC 3.23554^T^	China	OP546681	OP723490	OP672161	Li et al. (2022)
*Neopestalotiopsismusae*	MFLUCC 15-0776^T^	Thailand	NR156311	KX789685	KX789686	Norphanphoun et al. (2019)
*Neopestalotiopsisnatalensis*	CBS 138.41^T^	South Africa	NR_156288	KM199552	KM199466	Maharachchikumbura et al. (2014a)
*Neopestalotiopsisnebuloides*	BRIP 66617^T^	Australia	MK966338	MK977633	MK977632	Crous et al. (2020)
*Neopestalotiopsispaeonia-suffruticosa*	CGMCC 3.23555^T^	China	OP082292	OP204794	OP235980	Li et al. (2022)
*Neopestalotiopsispernambucana*	URM 7148-01^T^	Brazil	KJ792466	KU306739	N/A	Silvério et al. (2016)
*Neopestalotiopsisperukae*	FMBCC 11.3^T^	Pakistan	MH209077	MH523647	MH460876	Ul Haq et al. (2021)
*Neopestalotiopsispetila*	MFLUCC 17-1738^T^	Thailand	MK764276	MK764320	MK764342	Norphanphoun et al. (2019)
*Neopestalotiopsisphangngaensis*	MFLUCC 18-0119^T^	Thailand	MH388354	MH388390	MH412721	Tibpromma et al. (2018)
*Neopestalotiopsisphotiniae*	MFLUCC 22-0129^T^	China	OP498008	OP753368	OP752131	Sun et al. (2023)
*Neopestalotiopsisphyllostachydis*	CGMCC 3.23491^T^	China	OR247891	OR361423	OR381023	Razaghi et al. (2024)
*Neopestalotiopsispiceana*	CBS 394.48^T^	UK	KM199368	KM199527	KM199453	Maharachchikumbura et al.(2014b)
*Neopestalotiopsispsidii*	FMBCC 11.2^T^	Pakistan	MF783082	MH460874	MH477870	Ul Haq et al. (2021)
*Neopestalotiopsisrhizophorae*	MFLUCC 17-1551^T^	Thailand	MK764277	MK764321	MK764343	Norphanphoun et al. (2019)
*Neopestalotiopsisrhododendri*	GUCC 21504^T^	China	MW979577	MW980444	MW980443	Yang et al. (2021)
*Neopestalotiopsisrosae*	CBS 101057^T^	Zealand New	KM199359	KM199523	KM199429	Maharachchikumbura et al.(2014b)
*Neopestalotiopsisrosicola*	CFCC 51992^T^	China	KY885239	KY885243	KY885245	Norphanphoun et al. (2019)
*Neopestalotiopsissamarangensis*	MFLUCC 12-0233^T^	Thailand	JQ968609	JQ968611	JQ968610	Maharachchikumbura et al.(2014b)
*Neopestalotiopsissaprophytica*	MFLUCC 12-0282^T^	China	JX398982	JX399048	JX399017	Maharachchikumbura et al.(2014b)
*Neopestalotiopsisscalabiensis*	CAA1029^T^	Portugal	MW969748	MW959100	MW934611	Santos et al. (2022)
*Neopestalotiopsissiciliana*	AC46^T^	Italy	ON117813	ON107273	ON209162	Fiorenza et al. (2022)
*Neopestalotiopsissichuanensis*	CFCC 54338^T^	China	MW166231	MW199750	MW218524	Jiang et al. (2021)
*Neopestalotiopsissonneratiae*	MFLUCC 17-1745^T^	Thailand	MK764280	MK764324	MK764346	Norphanphoun et al. (2019)
*Neopestalotiopsissteyaertii*	IMI 192475^T^	Australia	KF582796	KF582792	KF582794	Maharachchikumbura et al.(2014a)
*Neopestalotiopsissubepidermalis*	CFCC 55160^T^	China	OK560699	OM622425	OM117690	Peng et al. (2022)
*Neopestalotiopsissuphanburiensis*	MFLUCC 22-0126^T^	Thailand	OP497994	OP753372	OP752135	Sun et al. (2023)
*Neopestalotiopsissurinamensis*	CBS 450.74^T^	Suriname	KM199351	KM199518	KM199465	Maharachchikumbura et al.(2014b)
*Neopestalotiopsisterricola*	CGMCC 3.23553^T^	China	OP082294	OP204796	OP235982	Li et al. (2022)
*Neopestalotiopsisthailandica*	MFLUCC 17-1730^T^	Thailand	MK764281	MK764325	MK764347	Norphanphoun et al. (2019)
*Neopestalotiopsisvacciniicola*	CAA1055^T^	Portugal	MW969751	MW959103	MW934614	Santos et al. (2022)
*Neopestalotiopsisumbrinospora*	MFLUCC 12-0285^T^	China	JX398984	JX399050	JX399019	Maharachchikumbura et al. (2012)
*Neopestalotiopsisvaccinii*	CAA1059 ^T^	Portugal	MW969747	MW959099	MW934610	Santos et al. (2022)
*Neopestalotiopsisvheenae*	BRIP 72293a^T^	Australia	MZ303792	MZ344177	MZ312685	Prasannath et al. (2021)
*Neopestalotiopsisvitis*	MFLUCC 15-1265^T^	China	KU140694	KU140676	KU140685	Jayawardena et al. (2016)
*Neopestalotiopsiszakeelii*	BRIP 72282a^T^	Australia	MZ303789	MZ344174	MZ312682	Prasannath et al. (2021)
*Neopestalotiopsiszimbabwana*	CBS 111495^T^	Zimbabwe	N/A	KM199545	KM199456	Maharachchikumbura et al.(2014b)
*Neopestalotiopsis* sp.	MEAN 1325	Portugal	MW794102	MW805414	MW802835	Diogo et al. (2021)
*Neopestalotiopsis* sp.	MEAN 1327	Portugal	MW794105	MW805416	MW802838	Diogo et al. (2021)
*Neopestalotiopsis* sp.	MEAN 1328	Spain	MW794115	MW805417	MW802848	Diogo et al. (2021)
*Neopestalotiopsis* sp.	PPS14	Peru	MK860757	MN000341	MN000344	Rodríguez-Gálvez et al. (2022)
*Neopestalotiopsis* sp.	PAK10	Peru	MK860755	MN000339	MN000342	Rodríguez-Gálvez et al. (2022)
*Neopestalotiopsis* sp.	PPS3	Peru	MK860756	MN000340	MN000343	Rodríguez-Gálvez et al. (2022)
*Neopestalotiopsis* sp.	CBS 664.94	Netherlands	KM199354	KM199525	KM199449	Maharachchikumbura et al.(2014b)
*Neopestalotiopsis* sp.	CBS 177.25	Unknown	KM199370	KM199533	KM199445	Maharachchikumbura et al.(2014b)
*Neopestalotiopsis* sp.	CFCC 54340	China	MW166235	MW199754	MW218528	Jiang et al. (2021)
*Neopestalotiopsis* sp.	ZX22B	China	MW166236	MW199755	MW218529	Jiang et al. (2021)
*Pestalotiopsiscolombiensis*	CBS 118553^T^	Colombia	KM199307	KM199488	KM199421	Maharachchikumbura et al.(2014b)
*Pestalotiopsisdiversiseta*	MFLUCC 12-0287^T^	China	NR_120187	JX399073	JX399040	Maharachchikumbura et al. (2012)
*Robillarda Africana*	CBS 122.75^T^	South Africa	KR873253	MH554414	MH554656	Crous et al. (2015a); Liu et al. (2019)
*Robillardaaquatic*	MFLUCC 21–0217^T^	Thailand	OL504777	N/A	N/A	Manawasinghe et al. (2022)
*Robillardaaustraliana*	CBS 143882^T^	Australia	MH554091	MH554525	MH554764	Liu et al. (2019)
***Robillardakhodaparastii***	**UJFCC2116^T^**	**Iran**	**PP038123**	**PP053743**	**PP053737**	**This study**
***Robillardakhodaparastii***	**UJFCC2117**	**Iran**	**PP038124**	**PP053744**	**PP053738**	**This study**
***Robillardakhodaparastii***	**UJFCC2118**	**Iran**	**PP038125**	**PP053745**	**PP053739**	**This study**
*Robillardamangiferae*	KUMCC 18-0180^T^	China	OL504777	N/A	N/A	Phookamsak et al. (2019)
*Robillardaroystoneae*	CBS 115445^T^	Hong Kong	KR873254	KR873310	KR873317	Crous et al. (2015a)
*Robillardasessilis*	CBS 114312^T^	Germany	KR873256	KR873312	KR873319	Crous et al. (2015a)
*Robillardaterrae*	CBS 587.71^T^	India	KJ710484	MH554493	MH554734	Crous et al. (2014); Liu et al. (2019)
*Robillarda* sp.	CPC 25020	N/A	KR873259	KR873315	KR873322	Crous et al. (2015a)

Maximum Likelihood (ML) analyses were performed using RAxML (Stamatakis 2006) as implemented in raxmlGUI 1.3 (Silvestro and Michalak 2012) with the ML + rapid bootstrap setting and the GTRGAMMA substitution model. A total of 1000 bootstrap replicates were conducted.

Maximum Parsimony (MP) analyses were performed with PAUP v. 4.0a169 (Swofford 2002). All molecular characters were treated as unordered and given equal weight, with gaps treated as missing data. The COLLAPSE command was set to MINBRLEN. MP analysis of the combined multilocus matrix was done using 1000 replicates of heuristic search with random addition of sequences, followed by TBR branch swapping (MULTREES option in effect, steepest descent option not in effect). Bootstrap analyses with 1000 replicates were performed similarly, with 10 rounds of random sequence addition and subsequent branch swapping during each bootstrap replicate. Bootstrap values ≤ 70% are considered low, those between 70% and 90% intermediate, and those ≥ 90% high.

## ﻿Results

### ﻿Molecular phylogeny

**Figure 1.**
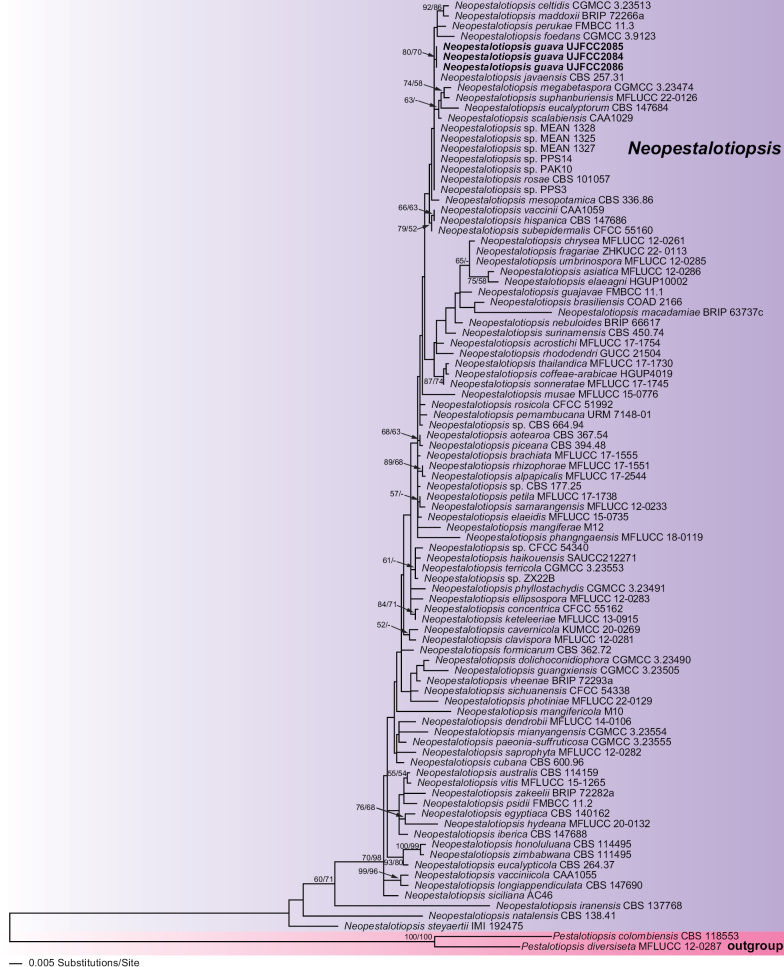
Phylogram of the best ML trees (lnL = −6,428.7652) revealed by RAxML from an analysis of the combined ITS–*tef1*–*tub2* matrix of selected *Neopestalotiopsis*. Strains in bold were sequenced in the current study. ML and MP bootstrap support above 50% are given at the first and second positions, respectively, above or below the branches.

In *Neopestalotiopsis*, of the 1363 characters included in the phylogenetic analyses (ITS-*tef1*-*tub2*), 255 were parsimony-informative (48 in ITS, 120 in *tef1*, and 87 in *tub2*). The phylogram of the best ML tree (lnL = −6,428.7652) obtained using RAxML is shown in Fig. 1. The MP analysis revealed 896 trees with a length of 805 (not shown) that had a similar topology to the ML tree (CI = 0.62, RI = 0.71, and RC = 0.37).

The isolates of *Neopestalotiopsis* from this study form a clade with a well-supported ML and MP BS (80/70%). Table 2 shows the base pair differences among other taxa that might be mistaken for the new species.

**Table 2:** Base pair differences between *Neopestalotiopsisguava* to related species in this study.

Species	Gene region		
	ITS	*tef1*	*tub2*
*Neopestalotiopsisceltidis*	4/434	10/432	5/371
*Neopestalotiopsiseucalyptorum*	1/505	10/475	1/340
*Neopestalotiopsisfoedans*	5/505	6/475	0/340
*Neopestalotiopsishispanica*	3/505	8/475	3/340
*Neopestalotiopsisjavaensis*	2/505	3/475	0/340
*Neopestalotiopsismaddoxii*	6/505	7/475	1/340
*Neopestalotiopsismegabetaspora*	2/434	11/432	2/371
*Neopestalotiopsismesopotamica*	2/505	9/475	1/340
*Neopestalotiopsisperukae*	5/505	1/475	1/340
*Neopestalotiopsisrosae*	2/505	7/475	0/340
*Neopestalotiopsisscalabiensis*	2/505	8/475	0/340
*Neopestalotiopsissubepidermalis*	3/456	8/432	3/371
*Neopestalotiopsissuphanburiensis*	2/434	8/411	2/371
*Neopestalotiopsisvaccinii*	2/505	8/475	3/340

**Figure 2.**
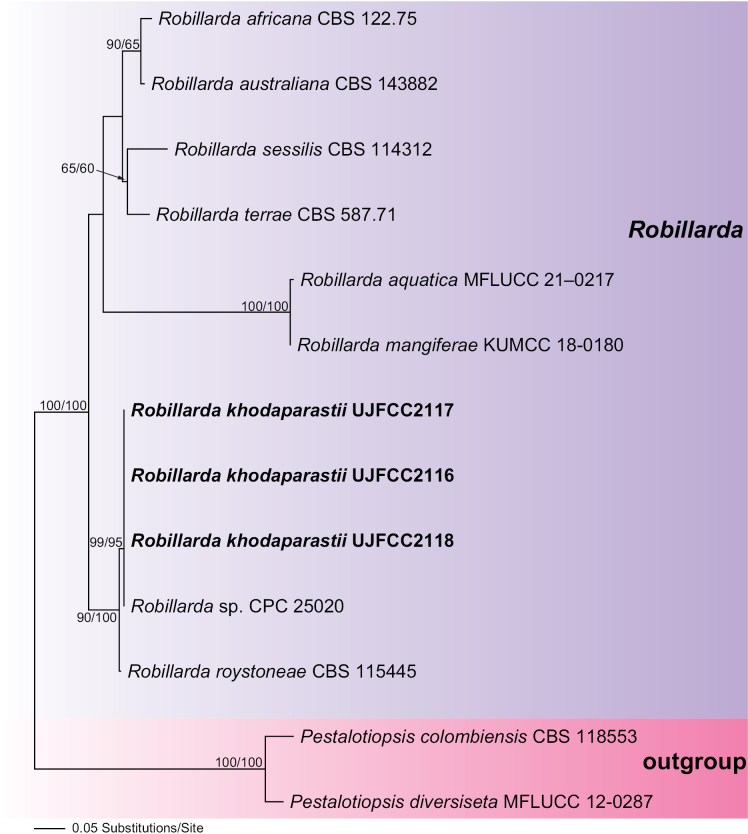
Phylogram of the best ML trees (lnL = −3,732.5074) revealed by RAxML from an analysis of the combined ITS–*tef1*–*tub2* matrix of selected *Robillarda* spp. Strains in bold were sequenced in the current study. ML and MP bootstrap support above 50% are given at the first and second positions, respectively, above or below the branches.

In *Robillarda*, of the 1306 characters included in the phylogenetic analyses (ITS-*tef1*-*tub2*), 238 were parsimony-informative (48 in ITS, 144 in *tef1*, and 46 in *tub2*). The phylogram of the best ML tree (lnL = −3,732.5074) obtained using RAxML is presented in Fig. 2. The MP analysis revealed a single tree with a length of 417 (not shown) that exhibited a similar topology to the ML tree (CI = 0.91, RI = 0.91, and RC = 0.08).

*Robillarda* isolates from the current study were grouped within a highly supported ML and MP bootstrap-supported clade, along with an unnamed isolate (CPC 25020). Analysis of these sequence data revealed identical sequences across all loci. This clade is a sister group of *R.roystoneae* (CBS 115445) with maximum ML and MP BS support. Molecularly, *R.khodaparastii* differs from *R.roystoneae* by 1 bp difference out of 532 bp in ITS, 2 bp differences out of 284 bp in *tef1* and 2 bp differences out of 237 bp in *tub2*. Based on these findings, we conclude that members of *Neopestalotiopsis* and *Robillarda* represent two independent, so far undescribed species.

### ﻿Taxonomy

#### ﻿ Neopestalotiopsis guava

Taxon classificationFungiAmphisphaerialesNeopestalotiopsis

A.R. Amirmijani, A. Pordel, & K. Dehghani sp. nov.

374DBF70-4A51-5044-8ADF-E1B3343A670E

85286

Fig. 3

##### Holotype.

Iran • Sistan and Baluchestan Province, Zar Abad region, from the infected leaves of *Psidiumguava*, 5 November 2021, leg. A. Pordel and A.R. Amirmijani (holotype: CUJ0100; ex-type culture: UJFCC2084).

##### Etymology.

Named after the host plant, *Psidiumguava*.

##### Description.

**Figure 3.**
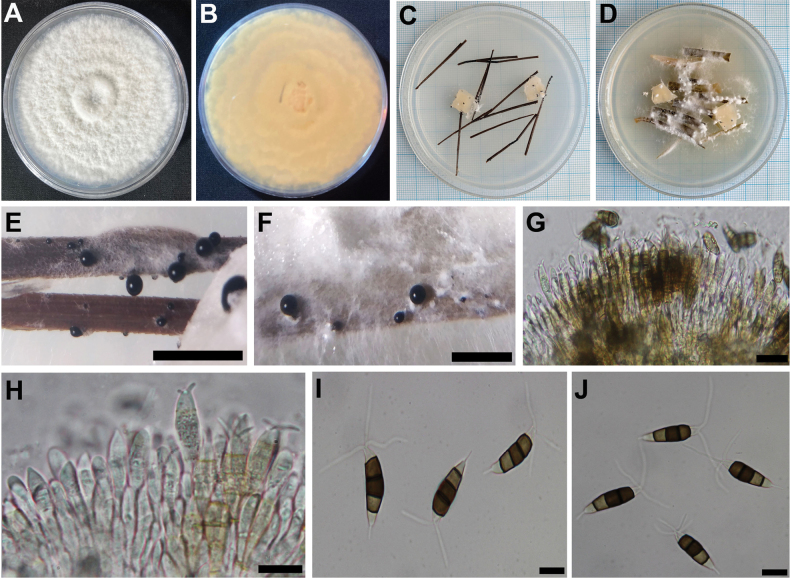
*Neopestalotiopsisguava* (Holotype UJFCC2084) **A, B** surface and reverse of colony after 7 days on PDA **C** colonies on PNA after 7 days **D** colonies on CLA after 7 days **E** conidiomata with black sporulation on PNA **F** conidiomata with black sporulation on CLA **G, H** conidiogenous cells **I, J** conidia with apical and basal appendage. Scale bars: 2 mm (**E, F**); 20 µm (**G, H**); 10 µm (**I, J**).

Conidiomata, solitary, black, and (300–)500–300(–700) m diam., and glistening conidial masses (Fig. 3E). Conidiogenous cells 6–10 × 2.5–4 µm, discrete, cylindrical, hyaline, smooth, thin-walled, simple, holoblastic-annelidic, percurrently, with collarette present. Conidia 18–30 × 5–7 µm, fusoid, four-septate, smooth, and slightly constricted at the septa; the basal cell thin walled, hyaline, 5–8 µm long; three median cells cylindrical, 15–18 µm long, smooth-walled, with septa darker than the rest of cell; the second cell from the base pale brown and 4–5 µm long; the third cell medium brown and 5 µm long; the fourth cell medium brown and 5 µm long; with septum between the third and fourth cell more distinct, broader, and darker brown than the other septa; the apical cell conic with a subacute apex, thin-walled, smooth, hyaline, 4–6 µm long, and with 2–3(–4) apical appendages (mostly 2) arising from the apical adage; apical appendages unbranched and straight, 11–24 µm long and up to 1 µm wide (n = 100); basal appendage single, filiform, unbranched, centric, 3–5 µm long, and up to 0.5 µm wide (n = 80).

##### Culture characteristics.

Colony on PDA attaining 70 mm diameter after 7 d at 25 °C, surface white and reverse pale cream (Fig. 3A, B), with fluffy white aerial mycelium, conidiomata scattered.

##### Other specimens examined.

Iran • Sistan and Baluchestan Province, Zar Abad region, from the infected leaves of *Psidiumguava*, 5 November 2021, leg. A. Pordel and A.R. Amirmijani (cultures UJFCC2084 and UJFCC2086).

##### Notes.

*Neopestalotiopsisguava* is phylogenetically closely related to a large clade containing *N.celtidis*, *N.ellipsospora*, *N.eucalyptorum*, *N.foedans*, *N.hispanica*, *N.javaensis*, *N.maddoxii*, *N.megabetaspora*, *N.mesopotamica*, *N.perukae*, *N.rosae*, *N.scalabiensis*, *N.subepidermalis*, *N.suphanburiensis* and *N.vaccinii*. However, distinct morphological differences distinguish it from all these species (Table 3).

**Table 3:** Morphological comparison of *Neopestalotiopsis* species related to this study.

Species	Conidial size (µm)	Apical appendages		Basal appendage	References
		Number	Length (µm)	Length (µm)	
*N.celtidis*	17.5–23.5 × 6–8	2–3(–4)	(7.5–)11.5–21(–25)	2–6	Razaghi et al. (2024)
*N.ellipsospora*	19–25 × 5–6.5	1–3	5–12	3–4	Maharachchikumbura et al. (2012)
*N.eucalyptorum*	(22.6)27.5–29.2(33.2) × (6.4)7.6–8.1(9.5)	3–4	(12.7)16.2–18.8(27.7)	(3.4)5.4–6.2(8.1)	Diogo et al. (2021)
*N.foedans*	19–24 × 5.7–6.9	2–3	6–18	3–6	Maharachchikumbura et al. (2012)
***N.guava***	**18–30 × 5–7**	**2–3(–4)**	**11–24 × 1–1.2**	**3–5 × 0.4–0.5**	**This study**
*N.hispanica*	(21.4) 22.9–24.1 (29.4) × (7.2)8.2–8.7(9.8)	2–3	(13)18.2–20.3(24.6)	(3.1)5.2–6.1(8.8)	Diogo et al. (2021)
*N.javaensis*	(24–)25–30(–31) × (6.5–)7–8.5(–9)	1–3	2–10(–18)	2–4	Maharachchikumbura et al. (2014b)
*N.maddoxii*	25–30 × 7–11	3	15–27	N/A	Prasannath et al. (2021)
*N.megabetaspora*	(19–)22–28 × 5.5–9	2–4	20–37	(2.5–)4.5–13	Razaghi et al. (2024)
*N.mesopotamica*	(25–)26–32(–34) × (7–)7.5–9(–9.5)	3–4	(25–)28–38(–41)	4–6.5	Maharachchikumbura et al. (2014b)
*N.perukae*	19.7 ± 1.4 × 6.4 ± 0.8	2	22.2 ± 5.8	3.8 ± 1.9	Ul Haq et al. (2021)
*N.rosae*	(20–)22–37(–29) × (7–)7.5–9.5(–10.5)	3–5	(22–)24–31(–33)	5–8	Maharachchikumbura et al. (2014b)
*N.scalabiensis*	(10.3–)13.8–15.1(–23.3) × (3.7–)4.8–6.6(–5.3)	2–3	5.9–31.8	N/A	Santos et al. (2022)
*N.subepidermalis*	(19.5–)20–25(–26) × 7.5–9(–9.5)	2–4	(26.5–)27–32.5(–33.5)	(6.5–)7–7.5(–8)	Peng et al. (2022)
*N.suphanburiensis*	19–29 × 4–7	2–3	9–21	2–11	Sun et al. (2023)
*N.vaccinii*	(11.0–)13.4–13.8(–15.2) × (4.9–)6.3–6.6(–7.5)	2–3	8.9–25.3	N/A	Santos et al. (2022)

#### ﻿ Robillarda khodaparastii

Taxon classificationFungiAmphisphaerialesNeopestalotiopsis

A.R. Amirmijani, A. Pordel & K. Dehghani sp. nov.

8DED929A-469C-540D-A306-E989D72AD67B

852862

Fig. 4

##### Holotype.

Iran • Sistan and Baluchestan Province, Ghasreghand Abad region, from the infected leaves of *Mangiferaindica*, 15 December 2021, leg. A. Pordel and A.R. Amirmijani (holotype: CUJ0103; ex-type culture: UJFCC2116).

##### Etymology.

The species name is suggested as a tribute to our professor, Dr. Seyed Akbar Khodaparast, in recognition of his significant contributions to the progress of mycology in Iran.

##### Description.

Sexual morph undetermined. Asexual morph coelomycetous. Conidiomata 300–340 mm diam., black, semi-immersed, solitary, scattered, irregular shape, glabrous, minutely ostiolate. Conidiophores are reduced to conidiogenous cells. Conidiogenous cells 13–20 × 3–6 µm, holoblastic, proliferation percurrent 1–3 times, discrete, subcylindrical to ampulliform, hyaline, aseptate, smooth-walled. Conidia 12–13 × 2–3 µm (n = 50), hyaline, cylindrical, straight, 1-septate, thin and smooth-walled, apical cell developed into a branched appendage; appendages 12–20 × 1–2.5 µm (n = 50), dividing into 2 branches, straight, non- flexuous, broadly tubular, narrower towards the apex.

##### Culture characteristics.

**Figure 4.**
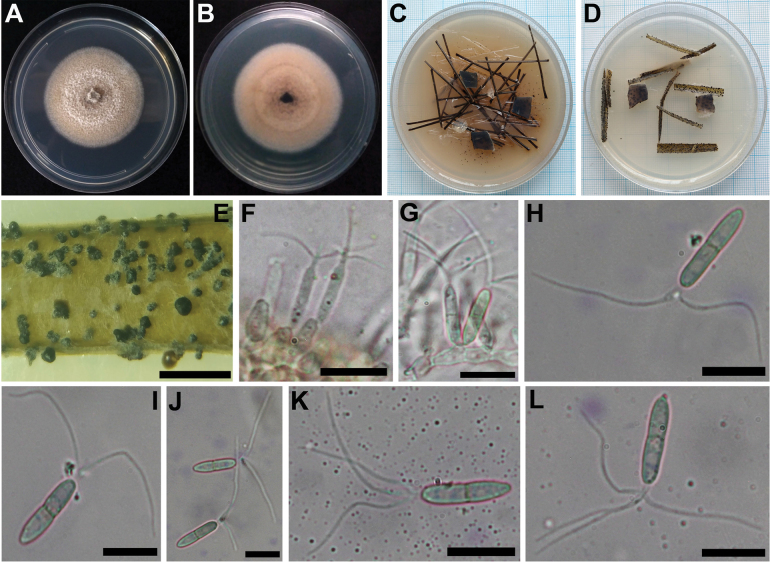
*Robillardakhodaparastii* (Holotype UJFCC2116) **A, B** surface and reverse of colony after 7 days on PDA **C** colonies on PNA after 7 days **D** colonies on CLA after 7 days **E** conidiomata with brown sporulation on CLA **F, G** conidiogenous cells **H–L** conidia with apical appendage. Scale bars: 2 mm (**E**); 10 µm (**F–L**).

Colony on PDA and MEA are similar, attaining 75 to 78 mm diameter after 7 d at 25 °C, surface and reverse white to cream (Fig. 4A, B), with fluffy white aerial mycelium, conidiomata scattered.

##### Other specimen examined.

Iran • Sistan and Baluchestan Province, Ghasreghand Abad region, from the infected leaves of *Mangiferaindica*, 15 December 2021, leg. A. Pordel and A.R. Amirmijani (cultures UJFCC2117 and UJFCC2118).

##### Notes.

A comparison of sequence data revealed complete identities among the *Robillarda* isolates. This clade was determined as a sister group of *R.roystoneae* (CBS 115445) with maximum ML and MP bootstrap support. *R.khodaparastii* is morphologically and phylogenetically near to *R.roystoneae*, but our species can be easily distinguished from the latter species by producing longer conidiogenous cells [13–20 × 3–6 vs. 7–12 × 2–3 µm] and shorter conidia [12–13 × 2–3 vs. (13–)14–15(–16) × 2.5–3(–3.5) µm].

## ﻿Discussion

Mango (*Mangiferaindica* L.) and Guava (*Psidiumguava* L.) are popular fruits in tropical and subtropical regions due to their delicious taste, high nutritional value, and economic importance in international markets (Kumar et al. 2021). The cultivation of mango has expanded beyond traditional and non-traditional production countries like the United Arab Emirates (UAE) (Saeed et al. 2017). Iran’s diverse climates, characterized by significant temperature variations between the northern and southern regions, allow for the production of various agricultural products (Saboki et al. 2012).

Several pestalotioid fungi have been reported from diverse hosts in Iran, including: *Pestalotiadisseminata*, *Pestalotiopsisacacia*, *P.biciliata*, *P.funereal*, *P.longiseta*, *P.longisetula*, *P.neglecta*, *P.nattrassii*, *P.trachycarpicola*, *P.vismiae*, *P.uvicola*, *Pseudopestalotiopsistheae*, and *Neopestalotiopsisasiatica*, *N.iranensis*, *N.mesopotamica* (Khodaparast and Hedjaroude 1994; Borhani and Mousazadeh 2004; Arzanlou et al. 2012; Naeimi et al. 2015; Ayoubi and Soleimani 2016a, 2016b; Atashi Khalilabad and Fotouhifar 2022; Bakhshi et al. 2022; Amirmijani et al. 2024).

According to Maharachchikumbura et al. (2011, 2013), many species of Pestalotioid fungi have been named based on their host associations due to the lack of reliable distinctive features. However, recent research has demonstrated that many introduced phylogenetic species within Pestalotioid fungi can be distinguished using combined sequence data from ITS-rDNA, β-tubulin (*tub2*), and tef-1α genes. In this study, we employed these gene sequences, along with morphological features, for phylogenetic analysis and species identification. As a result, we described two new species of pestalotioid fungi from Iran. These novel species contribute to a deeper understanding of the taxonomy and diversity of *Neopestalotiopsis* and *Robillarda* in Iran. However, it is likely that our findings represent only the tip of the iceberg.

These fungi were found on leaf spots of mango and guava in southern Iran. Generally, these genera are regarded as insignificant pathogens, however, they have been observed to cause diseases in various crops (Maharachchikumbura et al. 2012). These fungi often act as endophytes or saprobes, and their pathogenic roles still remain little studied and, therefore, inadequately understood. Although these species were isolated from leaf spots, we were unable to conduct pathogenicity tests in this study. Consequently, further research is necessary to evaluate their potential aggressiveness and negative effects on tropical fruits. Accurate species identification of plant-pathogenic genera (Jayawardena et al. 2015), such as Pestalotiopsis-like fungi, is crucial for plant pathologists in managing and controlling plant diseases. Ongoing studies aim to clarify the environmental factors contributing to leaf spot disease to develop effective control measures.

## Supplementary Material

XML Treatment for Neopestalotiopsis guava

XML Treatment for Robillarda khodaparastii

## ﻿Additional information

### Author ORCIDs

Amirreza Amirmijani https://orcid.org/0000-0002-4529-4415

Adel Pordel https://orcid.org/0000-0001-5999-9443

Kowsar Dehghani https://orcid.org/0009-0004-2930-3491

Mohammad Javad Pourmoghaddam https://orcid.org/0000-0001-6055-7503

Hans-Peter Grossart https://orcid.org/0000-0002-9141-0325

### Data availability

The data that support the findings of this study are available in GenBank (https://www.ncbi.nlm.nih.gov/genbank/) and in culture collections and fungal herbarium, as shown in Table 1 and the text.

## Supplementary materials

Supplementary material 1 Phylogenetic trees Data type rar Explanation note Single gene trees for all loci for both genera. https://binary.pensoft.net/file/1286263This dataset is made available under the Open Database License (http://opendatacommons.org/licenses/odbl/1.0/). The Open Database License (ODbL) is a license agreement intended to allow users to freely share, modify, and use this Dataset while maintaining this same freedom for others, provided that the original source and author(s) are credited.Amirreza Amirmijani, Adel Pordel, Kowsar Dehghani, Mohammad Javad Pourmoghaddam, Hossein Masigol, Hans-Peter Grossart
